# Supplementary material for: Constitutive expression of the transcriptional co-activator IκBζ promotes melanoma growth and immunotherapy resistance
Source: Nat Commun. 2025 Jun 25;16:5387. doi: 10.1038/s41467-025-60929-5 (PMC12198385; doi:10.1038/s41467-025-60929-5)
Supplement: Supplementary file 1 — Supplementary Information [file 41467_2025_60929_MOESM1_ESM.pdf]

Additional information

Supplementary Figure S1. Correlation of IκBζ with key driver mutations and transcription factor activation, and additional analyses on human melanoma patient samples.

Supplementary Figure S2. IκBζ target gene expression in additional melanoma cell lines.

Supplementary Figure S3. Additional data on IκBζ-dependent tumor cell proliferation.

Supplementary Figure S4. Additional data on IκBζ-dependent tumor cell growth.

Supplementary Figure S5. Additional data with patient material.

Supplementary Figure S6. Additional flow cytometry data from the B16-F10 mouse model.

Supplementary Figure S7. Additional data on IκBζ-dependent regulation of the transcription factor function of STAT1, STAT3, and p65.

Supplementary Figure S8. NF-κB and STAT do not mediate IκBζ-dependent gene repression of *Ccl5*, *Cxcl9*, and *Cxcl10*.

Supplementary Data 1. Analysis of IκBζ-regulated genes identified from the overlap of LOX-IMVI and D4M-3A cells in the RNA sequencing data.

Supplementary Data 2. Gene expression primer sequences.

Supplementary Data 3. ChIP primer sequences.

Supplementary Data 4. Summary of the clinical characteristics of human melanoma patients.

a

	Cell line	Primary vs metastasis	*BRAF*	*TP53*	*TERT*	*CDKN2A*	Other mutations
IκBζ-expressing cells	LOX-IMVI	metastasis	yes	yes	yes	no	*CCND1, NF1*
	SK-MEL-5	metastasis	yes	yes	yes	yes	
	SK-MEL-30	primary	no	yes	yes	yes	*NRAS*
	D4M-3A	primary	yes	no	no	no	*Pten*
	YUMM1.7	primary	yes	no	no	yes	*Pten*
IκBζ non-expressing cells	MV3	metastasis	no	no	no	no	
	MeWO	metastasis	no	yes	no	yes	*NF1, MAPK3*
	A375	primary	yes	no	yes	yes	
	UACC-62	primary	yes	no	no	no	*PTEN*
	UACC-257	primary	yes	yes	yes	yes	
	Malme-3M	metastasis	yes	yes	yes	yes	
	SK-MEL-3	metastasis	yes	yes	no	no	
	SK-MEL-2	metastasis	no	yes	yes	no	*NRAS*
	SK-MEL-28	primary	yes	yes	yes	no	*PTEN, CDK4*
	Mel2a	metastasis	yes	no	no	no	
	HMB-2	metastasis	yes	no	no	no	
	B16-F10	primary	no	no	no	yes	

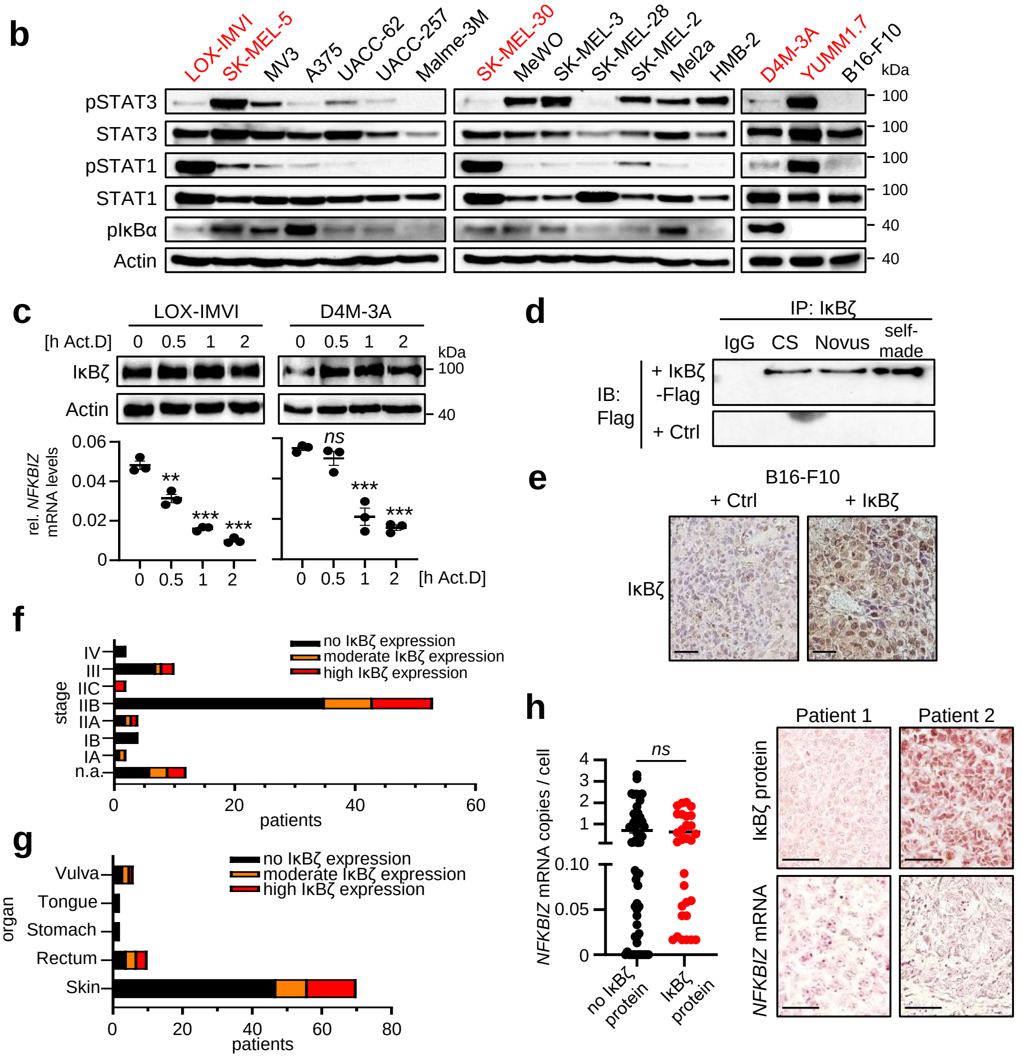

Supplementary Fig. 1 | Correlation of IκBζ with key driver mutations and transcription factor activation, and additional analyses on human melanoma patient samples. a Genomic characteristics of all used melanoma cell lines. **b** Immunoblot analysis of pSTAT3 (Y705), pSTAT1 (Y701) and pIκBα (S32) in all investigated melanoma cell lines at steady-state. **c** Effect of Actinomycin D (Act.D) on IκBζ protein and mRNA levels, normalized to β-Actin. Data represent the mean of 3 independent experiments ± standard deviation (SD). **d** Co-immunoprecipitation of human IκBζ in transfected HEK 293T cells. For immunoprecipitation (IP) of IκBζ, several antibodies were applied (CS = α-IκBζ from Cell Signaling; Novus = anti-IκBζ from Novus; self-made = self-made antibody raised against human IκBζ). Immunoblot detection (IB) was performed using a flag antibody. **e** IHC staining of human IκBζ in B16-F10 tumors harboring an empty control plasmid (Ctrl) or a human IκBζ overexpression. Scale: 50 µm. **f + g** Characteristics of the patient samples used for Fig. 1. **f** Stage of the disease. **g** Anatomical site. **h** Correlation of mRNA and protein levels of IκBζ in human melanoma samples. *NFKBIZ* mRNA levels were detected by RNAScope analysis and correlated to IκBζ protein expression using the same set of patient data. n = 57 patients with no IκBζ protein; n = 31 patients with IκBζ protein. Scale: 50 µm. Significance was calculated using a 2-tailed Student's t-test (**$p < 0.01$, ***$p < 0.001$, *ns* = not significant). Source data and exact p-values are provided in the Source Data file.

a

Term	Genes
Tumor progression and survival	*BCL3, EPAS1, TRIB2, IL6, IL11, DDIT4, STC1*
Immune evasion and inflammation	*CD274, CEBPD, IL6, IL11, SIGLEC15*
Interferon response	*IFIT2, IFIT3*
Metabolism	*SLC22A4, SLC2A6*
Angiogenesis	*CTSC, ENAH, ESM1, PTX3*
Epigenetics and chromatin regulation	*H2BC12*

b

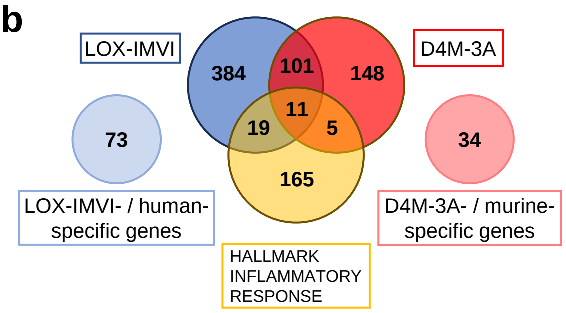

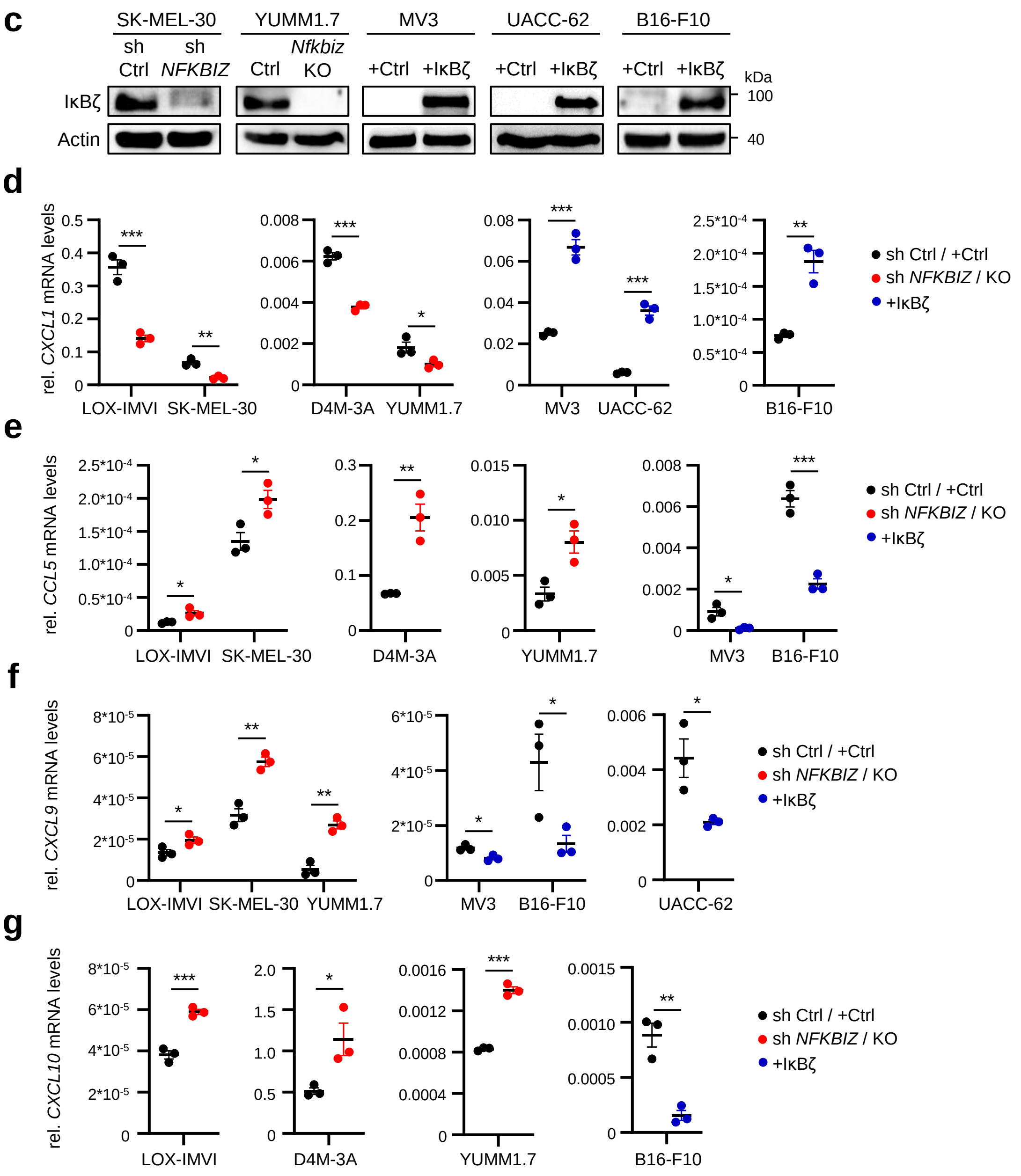

Supplementary Fig. 2 | IκBζ target gene expression in additional melanoma cell lines. a Gene set enrichment analysis of IκBζ-regulated genes identified from the overlap of LOX-IMVI and D4M-3A cells in the RNA sequencing data from Fig. 2d. **b** Comparison of IκBζ target genes encoding cytokines and chemokines in LOX-IMVI and D4M-3A cells with a previously published inflammatory gene set (GSEA term hallmark inflammatory response), using the same data as in Fig. 2d but applying a minimum fold change of 1.5 and $p \leq 0.1$. **c-g** *RPL37A* was used for normalization of all human gene expression data, and *Actb* was used to normalize gene expression data of murine cells. β-Actin served as a loading control for immunoblot analysis. **c** Validation of IκBζ knockdown or overexpression in various melanoma cell lines. **d** *CXCL1* mRNA levels. **e** *CCL5* mRNA levels. **f** *CXCL9* mRNA levels. **g** *CXCL10* mRNA levels. Shown is the mean of 3 independent experiments ± standard deviation (SD). Significance was calculated using a 2-tailed Student's t-test (*p < 0.05, **p < 0.01, ***p < 0.001). Source data and exact p-values are provided in the Source Data file.

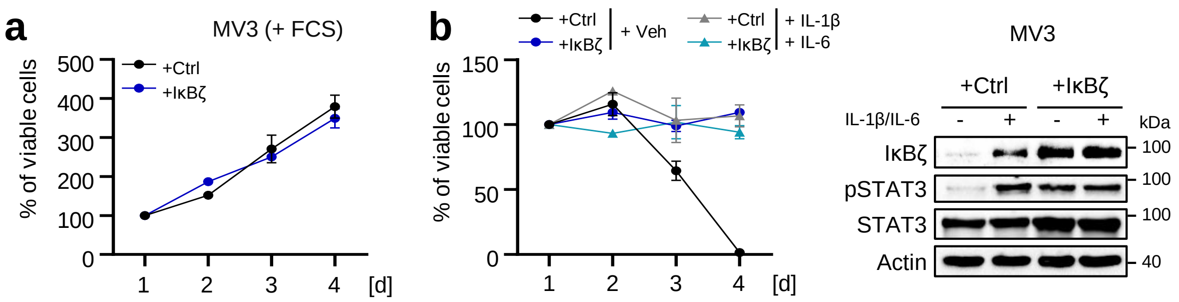

Supplementary Fig. 3 | Additional data on IκBζ-dependent tumor cell proliferation. a + b *In vitro* cell proliferation was assessed using the CellTiter-Glo assay (Promega). The calculation of the relative cell proliferation was done as described in Fig. 3. **a** Control or IκBζ-overexpressing MV3 cells were cultured in complete DMEM medium supplemented with 10 % FCS. **b** Same cells as in **a,** but cultured under starvation conditions (without FCS). Additionally, cells were treated with 100 ng/mL IL-1β and 100 ng/mL IL-6. IκBζ overexpression was controlled by immunoblotting, normalized to β-Actin levels. Shown is the mean of 3 independent experiments ± standard deviation (SD). Source data are provided in the Source Data file.

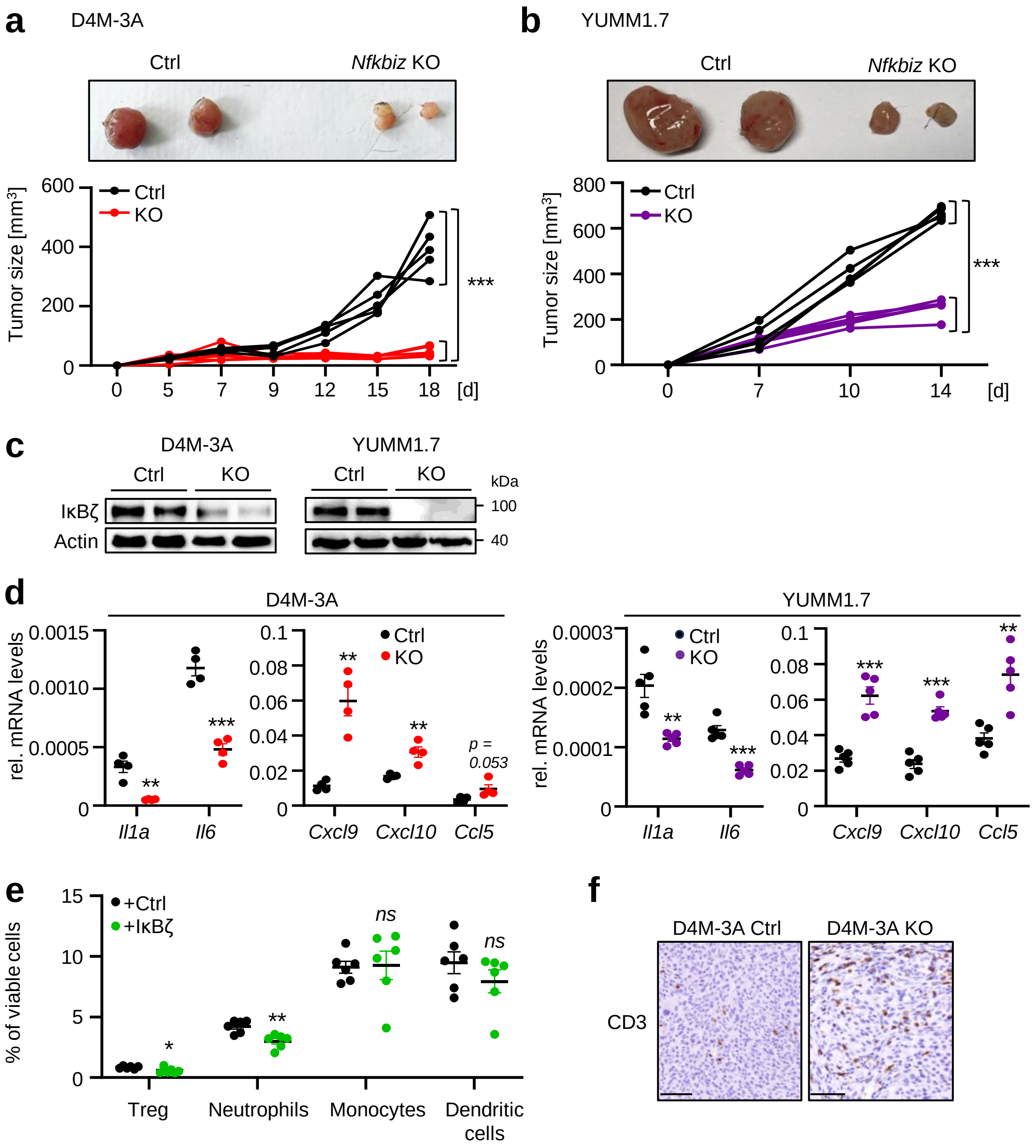

Supplementary Fig. 4 | Additional data on IκBζ-dependent tumor cell growth. a + b Tumor growth of control and IκBζ-depleted D4M-3A (**a**) and YUMM1.7 (**b**) cells, which were subcutaneously injected in the left and right flank of C57BL/6 mice. Tumor growth was assessed over 18 (D4M-3A) or 14 (YUMM1.7) days. n = 5. **c** IκBζ protein levels of tumors at the endpoint were analyzed by immunoblotting. β-Actin staining serves as a loading control. **d** Relative gene expression levels of IκBζ target genes in control and IκBζ-depleted D4M-3A and YUMM1.7 tumors at the endpoint. n = 4 (D4M-3A) or n = 5 (YUMM1.7). Relative mRNA levels were normalized to the reference gene *Actb*. **e** Flow cytometry analysis of infiltrating immune cells into control or IκBζ-overexpressing B16-F10 tumors. The following markers were applied on living (DAPI-negative) cells: regulatory T cells = $CD3^+$, $CD4^+$, $CD25^+$, Neutrophils = $Ly6G^+$, Monocytes = $Ly6C^+$ and dendritic cells = $CD11c^+$. n = 6. **f** Immunohistochemical staining of $CD3^+$ T cells in control or IκBζ-depleted D4M-3A tumors at the endpoint. Scale: 100 µm. Shown is the mean ± the standard error (SEM). Significance was calculated using a 2-tailed Student's t-test (*p < 0.05, **p < 0.01, ***p < 0.001, *ns* = not significant). Source data and exact p-values are provided in the Source Data file.

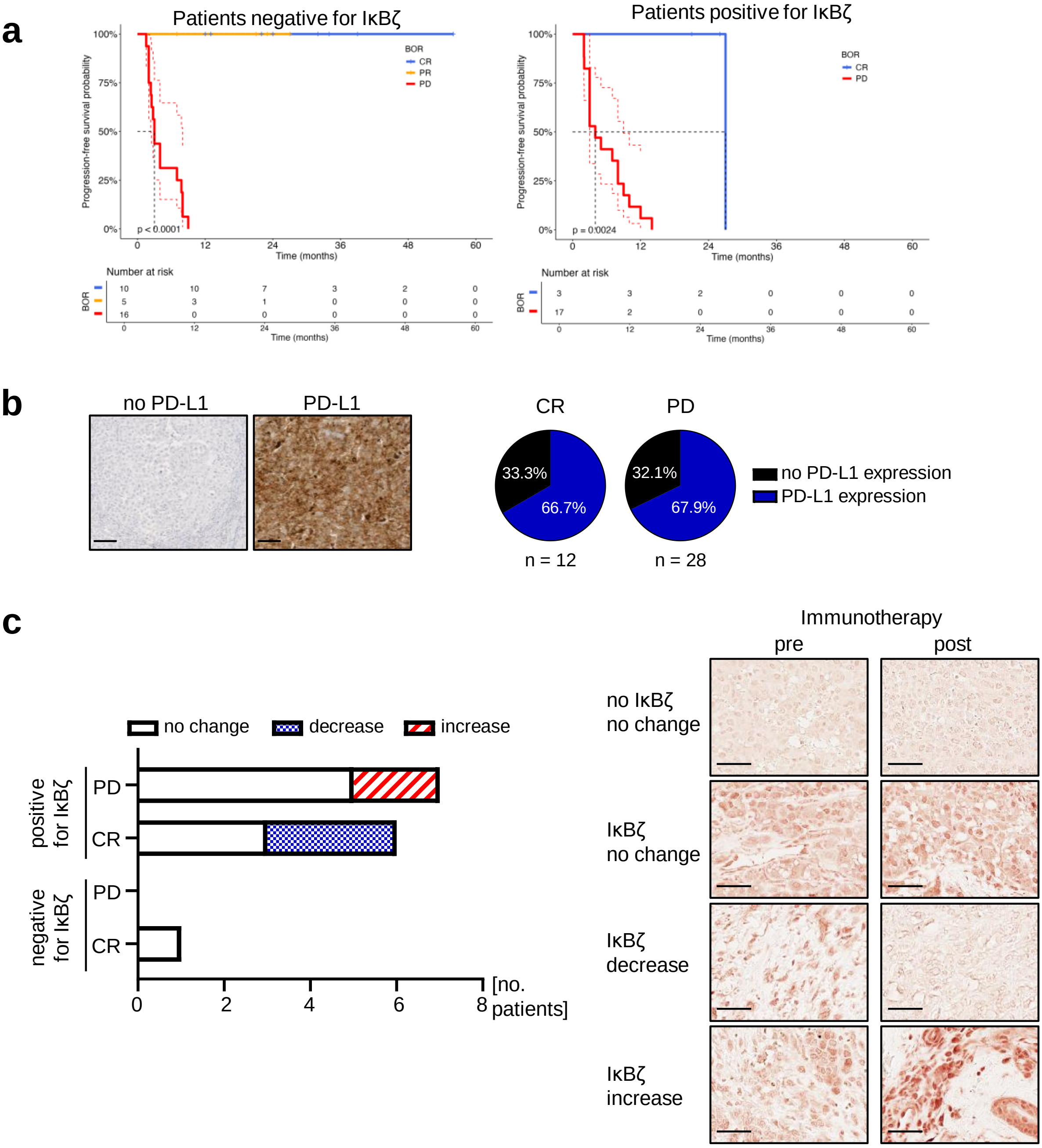

Supplementary Fig. 5 | Additional data with patient material. a Kaplan-Meier curve showing the progression-free survival (PFS) of immunotherapy-sensitive (CR and PR) and resistant (PD) patients, grouped according to the presence or absence of tumor-derived IκBζ protein expression. Shown is the median ± 95% CI. Two-tailed p-values were calculated and considered significant for $p < 0.05$. Survival analyses were conducted using the survminer R package (RStudio Version 1.3.1093). **b** α-PD-1L staining and correlation to immunotherapy responses in the same set of patients used in Fig. 5. **c** Analysis of IκBζ protein expression of melanoma patients pre- and post-immunotherapy. *Left:* overall evaluation of patients with positive or negative staining for IκBζ before treatment with immunotherapy, and further grouped into immunotherapy sensitive (CR) or resistant (PD) patients. White bars indicate no change in IκBζ protein levels after immunotherapy, blue-white indicates patients with decreased IκBζ protein levels after immunotherapy, and red-white displays patients with increased IκBζ expression levels post-immunotherapy. *Right:* Detection of IκBζ protein levels in 4 different patients pre- and post-immunotherapy. Scale: 50 µm. Source data are provided in the Source Data file.

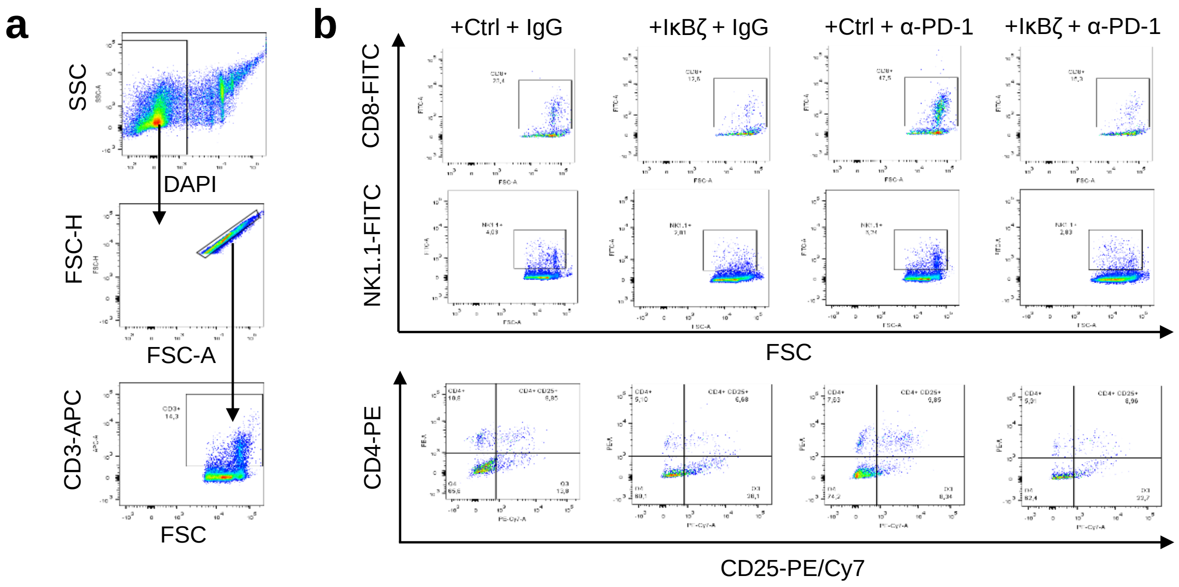

Supplementary Fig. 6 | Additional flow cytometry data from the B16-F10 mouse model. a Gating strategy used for the flow cytometry analysis. **b** Representative plots for the flow cytometric analysis of T cells and NK cells in IgG- or α-PD-1-treated control and IκBζ-overexpressing B16-F10 tumors at the endpoint.

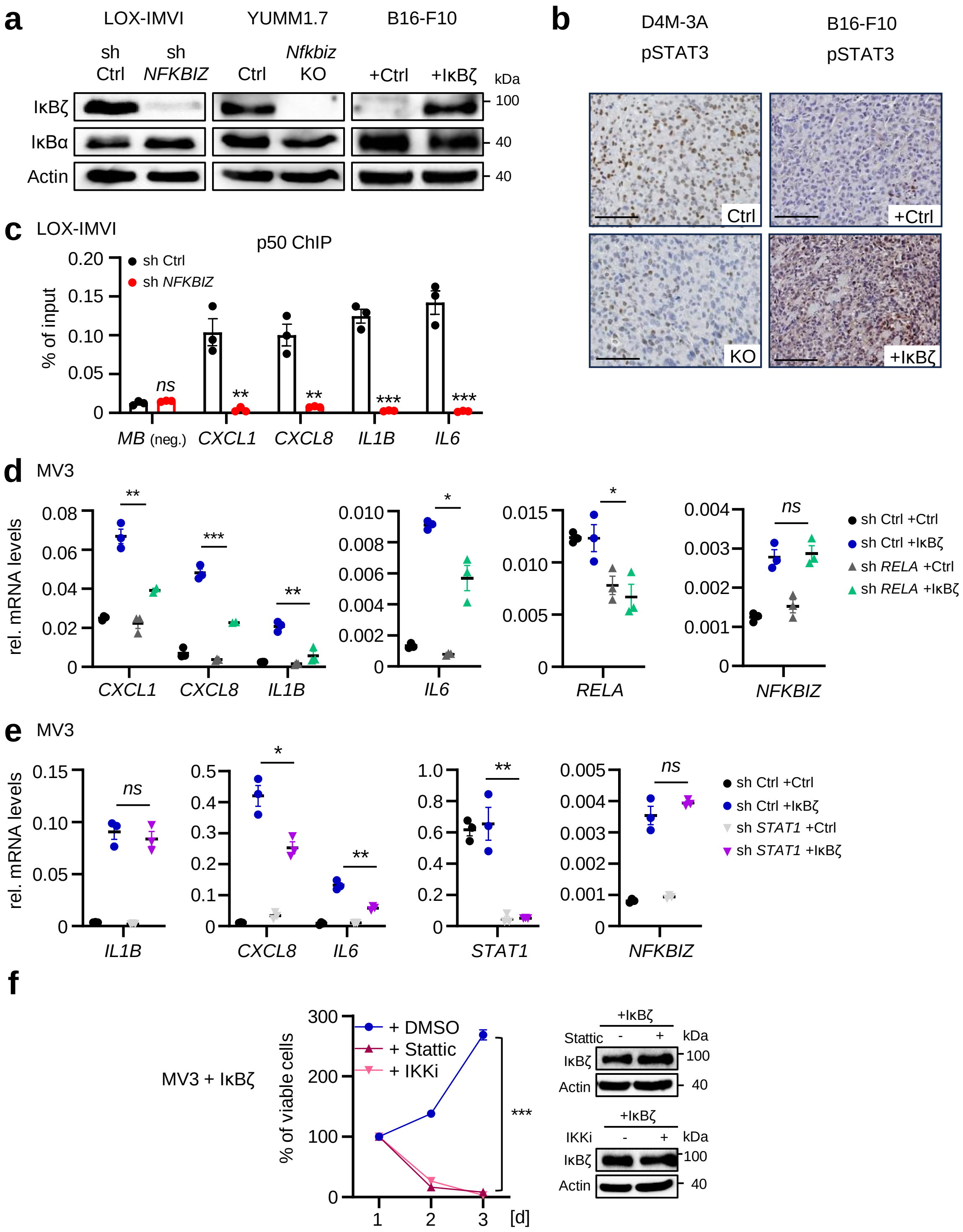

Supplementary Fig. 7 | Additional data on IκBζ-dependent regulation of the transcription factor function of STAT1, STAT3, and p65. a Immunoblot analysis of IκBα levels in IκBζ knockdown LOX-IMVI and D4M-3A cells, as well as IκBζ-overexpressing B16-F10 cells. β-Actin serves as a loading control. **b** IHC staining of phosphorylated STAT3 (Y705) in control or IκBζ-depleted D4M-3A tumors, and in control or IκBζ-overexpressing B16-F10 tumors at the experimental endpoint. Scale: 100 µm. **c** P50 chromatin immunoprecipitation in control and *NFKBIZ* knockdown LOX-IMVI cells. Shown is the relative percentage of input, *Myoglobin* (*MB*) serves as a negative control. **d + e** Gene expression of IκBζ target genes in control or IκBζ-overexpressing MV3 cells, in the presence or absence of **d** *RELA* or **e** *STAT1*. Both genes were lentivirally knocked down using shRNA; control cells were generated using non-coding shRNA (sh Ctrl). Subsequently, empty plasmid or IκBζ was transiently overexpressed. Shown are relative mRNA levels, normalized to the reference gene *RPL37A*. **f** Cell viability assay of IκBζ-overexpressing MV3 cells, treated with the IKK inhibitor IMD-0354 (5 µM) to inhibit NF-κB or Stattic (10 µM) to suppress STAT1 and STAT3 activation. Cell proliferation was assessed using the CellTiterGlo assay from Promega. Shown is the mean of 3 independent experiments ± standard deviation (SD). Significance was calculated using a 2-tailed Student's t-test (*p < 0.05, **p < 0.01, ***p < 0.001, *ns* = not significant). Source data and exact p-values are provided in the Source Data file.

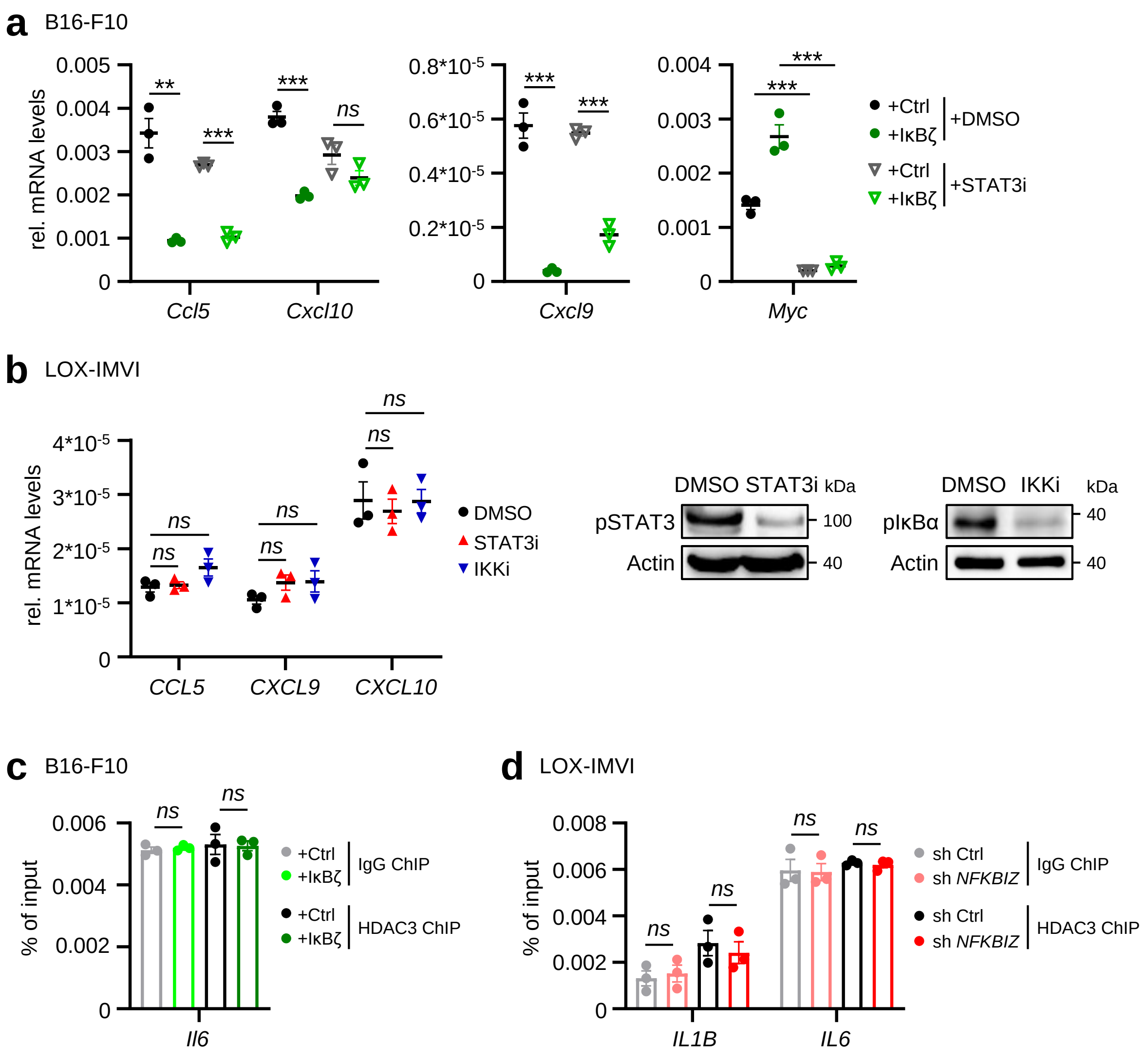

Supplementary Fig. 8 | NF-κB and STAT do not mediate IκBζ-dependent gene repression of *Ccl5*, *Cxcl9*, and *Cxcl10*. a Control and IκBζ-overexpressing B16-F10 cells, treated for 24 hours with 10 µM STAT1/3 inhibitor Stattic. Relative gene expression was normalized over *Actb*. *Myc* served as an inhibitor control. **b** LOX-IMVI cells treated for 24 hours with 5 µM IKK inhibitor IMD-0354 (IKKi) or 10 µM STAT1/3 inhibitor Stattic (STAT3i). *Left:* Relative gene expression levels normalized to *RPL37A*. *Right:* Inhibition control for phosphorylated STAT3 (pSTAT3 Y705) and NF-κB (phosphorylated IκBα (S32). β-Actin is a loading control. n = 3. **c** Additional data of the ChIP assay in control and IκBζ-overexpressing B16-F10 cells shown in Fig. 9d. **d** Additional data of the ChIP assay in shRNA control and *NFKBIZ* knockdown LOX-IMVI cells shown in Fig. 9e. Shown is the mean of 3 independent experiments ± standard deviation (SD). Significance was calculated using a 2-tailed Student's t-test (**p < 0.01, ***p < 0.001, *ns* = not significant). Source data and exact p-values are provided in the Source Data file.
