# Supplementary material for: Constitutive expression of the transcriptional co-activator IκBζ promotes melanoma growth and immunotherapy resistance
Source: Nat Commun. 2025 Jun 25;16:5387. doi: 10.1038/s41467-025-60929-5 (PMC12198385; doi:10.1038/s41467-025-60929-5)
Supplement: Supplementary file 2 — Description of Additional Supplementary Files [file 41467_2025_60929_MOESM2_ESM.pdf]

Description of Additional Supplementary Files

File Name: Supplementary Data 1
Description: Analysis of IκBζ-regulated genes identified from the overlap of LOX-IMVI and D4M-3A cells in the RNA sequencing data.

File Name: Supplementary Data 2
Description: Gene expression primer sequences.

File Name: Supplementary Data 3
Description: ChIP primer sequences.

File Name: Supplementary Data 4
Description: Summary of the clinical characteristics of human melanoma patients.
